# Gene Network Landscape of the Ciliate *Tetrahymena thermophila*

**DOI:** 10.1371/journal.pone.0020124

**Published:** 2011-05-26

**Authors:** Jie Xiong, Dongxia Yuan, Jeffrey S. Fillingham, Jyoti Garg, Xingyi Lu, Yue Chang, Yifan Liu, Chengjie Fu, Ronald E. Pearlman, Wei Miao

**Affiliations:** 1 Key Laboratory of Aquatic Biodiversity and Conservation, Institute of Hydrobiology, Chinese Academy of Sciences, Wuhan, China; 2 Graduate School of Chinese Academy of Sciences, Beijing, China; 3 State Key Laboratory of Freshwater Ecology and Biotechnology, Institute of Hydrobiology, Chinese Academy of Sciences, Wuhan, China; 4 Department of Chemistry and Biology, Ryerson University, Toronto, Ontario, Canada; 5 Department of Biology and Center for Research in Mass Spectrometry, York University, Toronto, Ontario, Canada; 6 Pathology Department, University of Michigan Medical School, Ann Arbor, Michigan, United States of America; University of Minnesota, United States of America

## Abstract

**Background:**

Genome-wide expression data of gene microarrays can be used to infer gene networks. At a cellular level, a gene network provides a picture of the modules in which genes are densely connected, and of the hub genes, which are highly connected with other genes. A gene network is useful to identify the genes involved in the same pathway, in a protein complex or that are co-regulated. In this study, we used different methods to find gene networks in the ciliate *Tetrahymena thermophila*, and describe some important properties of this network, such as modules and hubs.

**Methodology/Principal Findings:**

Using 67 single channel microarrays, we constructed the *Tetrahymena* gene network (TGN) using three methods: the Pearson correlation coefficient (PCC), the Spearman correlation coefficient (SCC) and the context likelihood of relatedness (CLR) algorithm. The accuracy and coverage of the three networks were evaluated using four conserved protein complexes in yeast. The CLR network with a Z-score threshold 3.49 was determined to be the most robust. The TGN was partitioned, and 55 modules were found. In addition, analysis of the arbitrarily determined 1200 hubs showed that these hubs could be sorted into six groups according to their expression profiles. We also investigated human disease orthologs in *Tetrahymena* that are missing in yeast and provide evidence indicating that some of these are involved in the same process in *Tetrahymena* as in human.

**Conclusions/Significance:**

This study constructed a *Tetrahymena* gene network, provided new insights to the properties of this biological network, and presents an important resource to study *Tetrahymena* genes at the pathway level.

## Introduction

High throughput gene expression data as generated by DNA microarray technology provides insight into the behavior of individual genes under various conditions [1]. The microarray expression levels under different physiological states constitute an expression profile of each gene, which can be used in genome-wide exploration and analysis of coexpression patterns and construction of gene networks [2]. Gene networks characterize the interactions of bio-molecules such as the physical interactions, metabolite flow, regulatory relationships, co-expression relationships, and more [3]. Network analysis can be used to identify related biological processes or pathways at the cellular level, which are manifested in the form of modules in the gene network. The module, representing a cluster of genes which are tightly joined together and between which there are only sparse connections, is an important property of a gene network [4]. In addition, the hub that represents the genes highly connected with others in a network, is also an important property of a scale free network and is of great biological significance [5].

Many methods such as the correlation coefficients [6], mutual information [7], [8] and reverse engineering [9], [10] have been used to infer gene networks for large scale expression data in diverse organisms such as the yeast *Saccharomyces cerevisiae* [6], [11], *Arabidopsis* [12], [13], human [14], [15], the parasite *Plasmodium falciparum* [16] and the fungus *Aspergillus niger* [17]. *Tetrahymena thermophila* is a protist, a free-living ciliated protozoan widely distributed in freshwater environments around the world [18], and is a useful and well studied model organism for molecular and cellular biology [19]. *Tetrahymena* has two distinct nuclei which separate germline and soma functions within a single cell. The diploid germline-like micronucleus (MIC) is the storehouse of genetic information that is passed on to sexual progeny. The polyploid soma-like macronucleus (MAC) is actively transcribed during vegetative proliferation and determines the phenotype of the cell. The structural and functional complexity of a *Tetrahymena* cell is equal to or greater than that of human and other metazoan cells. Studies on *Tetrahymena* have led to the development of a number of important seminal paradigms and numerous scientific breakthroughs [20], [21], [22]. In addition, a number of molecular genetic technologies and genomic resources have been developed [23], [24].

In 2009, Miao et al. reported the first microarray platform of the AT-rich *Tetrahymena* genome based on 50 microarrays of RNA expressed at different stages of the *Tetrahymena* life cycle [25]. Here we describe a *Tetrahymena* gene network (TGN) using these and 17 additional arrays. Three methods were adopted for this analysis, including the Pearson correlation coefficient (PCC), Spearman correlation coefficient (SCC) and the context likelihood of relatedness (CLR) algorithm [26]. The performances of these three methods were compared to determine the TGN. Two important properties, modules and hubs, were investigated in the TGN. Coupled with an analysis of orthologs to genes involved in human diseases, this work provides a valuable resource for future investigations of important biological processes and pathways in *Tetrahymena* and their relationships to human illness.

## Results

### Constructing a *Tetrahymena* Gene Network

Sixty-seven Roche NimbleGen single channel microarray samples were analyzed. After gene filtering, three methods were used to construct gene networks: the Pearson and Spearman correlation coefficient, and the mutual information based context likelihood of relatedness (CLR) algorithm [26]. The modules and hubs were determined from the global network. The biological function categories of these modules and hubs were analyzed using the gene ontology (GO) approach [27]. In addition, analysis of the *T. thermophila* macronuclear genome sequence has identified 58 *Tetrahymena* orthologs of human disease genes that are missing in yeast [19], and we also focused our analysis on these genes.

**Figure 1:**
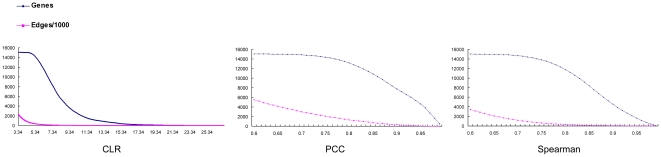
The node and edge number against cutoff values for three methods. For the CLR method, the cutoff value represents the Z-score with a minimal value of 3.34 corresponding to the 60% confidence level of the FDR test; for the PCC and SCC methods, the cutoff value represents the correlation coefficient. The minimal correlation coefficient is 0.6.

**Figure 2:**
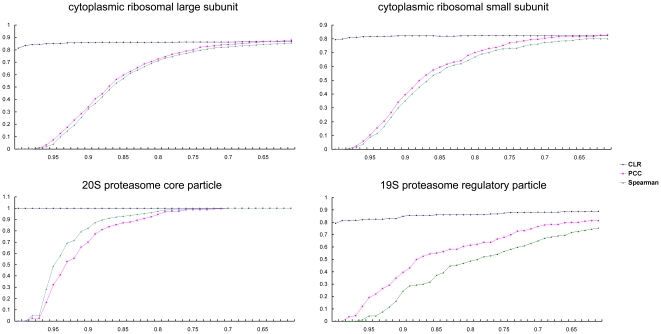
Overall performance of three methods for four protein complexes. The F-score against the cutoff values (X-axis) of three methods for each protein complex is presented. Blue, CLR method; Pink, PCC method; Green, SCC method. For the CLR method, the cutoff value means the different confidence levels of the FDR test; for the PCC and SCC methods, the cutoff values represent the correlation coefficient.

The correlation coefficient was used as the cutoff value for Pearson and Spearman correlation methods, and the Z-score was used for the CLR method. The number of nodes (genes) and edges (interactions of one gene to another determined by threshold) computed using different methods are shown in Figure 1. With increasing correlation coefficients or Z-score, both the node and edge number decreased. However, as the cutoff reached a relatively high value, the decrease in edge values became slower than that of nodes, leading to an increase in the network density. As shown in Figure 1, 0.6 was used as the minimal cutoff value for the two correlation methods and 3.34 (corresponding to 60% confidence level in the FDR test) was used as the minimal cutoff Z-score for the CLR method. Under these minimal values, the networks of the three methods contained about the same number of nodes (Figure 1), however, the edge numbers of these three methods were very different. For the two correlation methods, the edge number for the Pearson method was greater than the Spearman method with the same accuracy, suggesting a higher false positive rate for the PCC method. However, the PCC and the SCC methods were 2.4 times and 1.5 times respectively the edge number as those of the CLR method. This indicates that the CLR method may have higher prediction accuracy than the two correlation methods. To verify this and choose an appropriate cutoff, we selected four yeast protein complexes and identified the one to one orthologs between yeast and *T. thermophila*. The cytoplasmic ribosomal large subunit, cytoplasmic ribosomal small subunit, 20S proteasome core particle and the 19S proteasome regulatory particle, were used as benchmarks to determine the best of these three methods and the appropriate cutoff value. Using these four complexes, the accuracy (p value), the coverage (r value) and the overall performance (F-score) (see Methods) were calculated and are shown in Figure 2 and Figure S1. Comparing the three methods, the F-score, accuracy and coverage of CLR is consistently better than those of the other methods, especially for the 19S proteasome regulatory particle complex which contained 19 orthologous genes. Seventeen genes were shown to exist in a *Tetrahymena* proteasome complex by mass spectrometry (see below, Module-19). It is worth noting that the PCC and SCC networks would have to be two times larger than the CLR network (data not shown) for getting the same accuracy and coverage, so the specificity of the CLR method is also better than the correlation coefficient methodology. Based on the above results, CLR was used as the method of choice. For presentation of CLR gene network data, the X-axis represents the FDR test confidence level. It has been reported that the CLR algorithm performed best at 60% confidence level [26]. In our study, the four complexes analyzed showed that the appropriate threshold is 77% for the cytoplasmic ribosomal large subunit, 81% for the cytoplasmic ribosomal small subunit, 99% for the 20S proteasome core particle and 86% for the 19S proteasome regulatory particle. Taking into account the accuracy and coverage, 77%, corresponding to a Z-score of 3.49, was used as the cutoff confidence level. At this threshold, the CLR network possessed 15,049 nodes and 1,958,477 edges, and is considered the TGN.

### Functional modules of the *Tetrahymena* life cycle

We used the MCL algorithm to partition TGN into gene modules. The MCL algorithm is a fast and efficient clustering algorithm [28] that has been extensively applied in many studies, such as the yeast protein interaction network [29], protein family networks [28], a human coexpression network [15], and an *Arabidopsis* gene coexpression network [30]. After MCL clustering, 55 modules (modules 1–55) were found for the TGN. To investigate the functions of these modules, we performed an enrichment analysis of biological process GO terms for 21 of the 55 modules with more than 100 genes. Data for these 21 modules are presented in Table 1. Analysis of several modules is presented below:

**Table 1:** Detailed information of 21 modules containing more than 100 genes in the TGN.

Module	Gene NO	GO ANNO NO	GO ANNO PER	BP ANNO NO	Ortho No	Ortho PER
Module-1	3533	1287	36.43%	795	741	20.97%
Module-2	1703	790	46.39%	548	605	35.53%
Module-3	1369	510	37.25%	327	266	19.43%
Module-4	988	271	27.43%	156	278	28.14%
Module-5	929	314	33.80%	196	179	19.27%
Module-6	836	287	34.33%	154	114	13.64%
Module-7	827	365	44.14%	233	336	40.63%
Module-8	824	156	18.93%	132	29	3.52%
Module-9	583	264	45.28%	152	307	52.66%
Module-10	565	202	35.75%	124	139	24.60%
Module-11	338	134	39.64%	86	71	21.01%
Module-12	332	93	28.01%	52	53	15.96%
Module-13	314	65	20.70%	39	27	8.60%
Module-14	313	87	27.80%	61	8	2.56%
Module-15	260	106	40.77%	64	45	17.31%
Module-16	181	82	45.30%	49	58	32.04%
Module-17	162	93	57.41%	56	89	54.94%
Module-18	127	35	27.56%	19	22	17.32%
Module-19	117	77	65.81%	64	77	65.81%
Module-20	114	18	15.79%	9	11	9.65%
Module-21	109	38	34.86%	25	22	20.18%

The modules are named by the gene numbers in descending order. GO ANNO NO, indicates the number of GO annotated genes; GO ANNO PER, indicates the percentage of GO annotated genes; BP, biological process; Ortho, orthologs with other eukaryotes (see Materials and Methods).

**Module-1** is the largest module partitioned by the MCL method. It has 3533 genes, and 36.43 percent (1287 genes) are annotated by GO terms. Genes in this module are significantly over-represented in various functions (Table S1). For these enrichment terms, 475 of 795 genes (59.7%), are annotated by the GO term of macromolecule metabolic process.

Within Module-1, some enriched processes include some genes important for *Tetrahymena* conjugation. For example, the term “establishment or maintenance of chromatin architecture” includes genes Pdd1 and Pdd3. Nuclear dimorphism in *Tetrahymena* identifies specific features in conjugation. During MAC differentiation, several types of developmentally programmed DNA rearrangements occur [31]. One such rearrangement is the deletion of segments of the MIC genome known as internally eliminated sequences (IESs). A number of genes have been shown to be involved in programmed DNA elimination, such as *Tw*i1 [32], *Dcl1* [33], *Pdd1* and *Pdd3* [34], *CnjB* [35], *Ema1* [36], *Giw1* [37], *Ezl1* [38], *Hen1* [39], *Tpb2* [40], and *Die5* [41]. We have inspected these 11 genes in our network, and found they are closely related to each other with high Z-scores (Figure S2). In addition, there are 147 genes each connected to 11 genes (data not shown). The extracted sub-network of these 158 genes (147 plus 11 genes) shows a high edge-node ratio (network density) of 77 (Figure S2), suggesting that the network consisting of these genes are good candidates to be involved in DNA rearrangement during conjugation in *Tetrahymena*.

**Module-2** contains 1703 genes with 46.4 percent annotated by GO terms. For this module, a significant overrepresentation of genes are involved in oxidative phosphorylation (Table S1), represented by GO terms such as oxidation reduction, hydrogen transport, oxygen and reactive oxygen species, metabolic process, and transmembrane ion transport. In the oxidative phosphorylation pathway, there are five complexes, including the NADH-coenzyme Q oxidoreductase (complex I), succinate-Q oxidoreductase (complex II), Q-cytochrome c oxidoreductase (complex III), and cytochrome c oxidase (complex IV), and the electron transfer and the ATP synthase (complex V). It has been reported that there is a special F_o_ sector of ATP synthase in *Tetrahymena* and even in the alveolate group [42]. In that study, 89 proteins were identified in the ATP synthase complex using mass spectrometric analysis. For the 89 proteins, 8 were encoded in the mitochondrial genome and were not included in the microarray data, and 79 genes appeared in our TGN. We extracted the subnetwork of these 79 genes, and found that 71 were densely connected (Figure S3). This result suggests the high reliability of the TGN analysis. In addition, another 66 genes were found to interact with at least 60 genes of the 71 genes densely connected genes described above (Figure S3), which suggests that there are other genes associated with this protein complex.

Another set of genes overrepresented in this module is involved in glycolysis and related pathways, such as the citric acid (TCA) cycle, the pentose phosphate pathway, starch and sucrose metabolism, pyruvate metabolism, and propanoate metabolism (Table S1) pathways involved in energy (ATP) and reducing power (NADH) production [43], [44]. Module-2 is closely related to energy metabolism of *Tetrahymena.*

**Figure 3:**
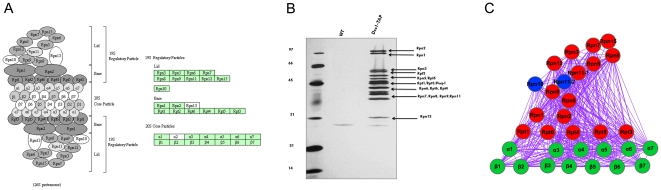
Possible components of the *Tetrahymena* proteasome complex. A, KEGG annotated *Tetrahymena* proteasome complex (http://www.kegg.jp/kegg-bin/show_pathway?tet03050); B, Silver stained gel of a pull down experiment using Dss1 (Rpn15) as bait that identifies proteins of the *Tetrahymena* proteasome; C, The network of the possible *Tetrahymena* proteasome complex.

**Module-19** contains 117 genes with the highest GO annotated percent (65.8 %) and the highest percent of orthologs (also 65.8 %) with other eukaryotes (Table 1). GO enrichment analysis shows that proteins encoded by genes in this module are significantly involved in proteolysis with GO terms including proteolysis involved in cellular protein catabolic process (GO: 0051603) and regulation of protein metabolic process (GO: 0051246), (Table S1). Comparison to the KEGG pathway also shows that this module contains a majority of genes in the proteasome complex (KEGG pathway: tet03050). The main function of the proteasome is to degrade unneeded or damaged proteins by proteolysis, and the complex is part of a major mechanism by which cells regulate the concentration of particular proteins and degrade misfolded proteins [45]. The most common form is the 26S proteasome containing the 20S core particle and the 19S regulatory particle (Figure 3-A). Using immunopurification and mass spectrometry, we have identified 17 proteins in the 19S regulatory particle (Figure 3-B). Sixteen of these 17 genes are densely connected, and the other gene (TTHERM_01014660, a homolog of Rpt2) was mispredicted by the gene model as shown by our RNA-Seq data (unpublished data) (Figure S4), which caused an incorrect normalization for the expression value in the microarray data. Using the 16 genes as the bait (Figure 3-C, red nodes), we find that each of the 13 genes (Figure 3-C, green nodes) in the 20S core particle (annotated by the KEGG pathway) is connected to at least 13 genes in the bait. Again, setting the 29 genes (16 genes of the 19S regulatory particle and 13 genes of the 20S core particle) as bait, we find that the other two KEGG annotated genes, TTHERM_00471830 (a homolog of Rpn10) and TTHERM_00476810 (another homolog of Rpn11 different from the pull down experiment), are also densely connected to the 29 bait genes (Figure 3-C, blue nodes), suggesting these two genes as possible components of the *Tetrahymena* 26S proteasome. In addition, two ubiquitin-associated genes (TTHERM_00471920 and TTHERM_00355130) in Module-19 are also densely connected to the proteasome complex, indicating that these two genes may function in proteolysis processes.

**Module-8, -13, -14 and -20** have a low GO annotated percent (range from 15.8% to 27.8%) and a low orthologs percent (range from 2.6% to 9.7%) with other eukaryotes (Table 1). In addition, only a few KEGG annotated pathway genes are found in these modules. Based on these data, these four modules should be more representative of unique biological functions after the divergence of the oligohymenophorean ciliates (e.g. *Paramecium* and *Tetrahymena*, about 800 Mya). Since few *Paramecium* orthologs are found, these four modules are possibly unique in *Tetrahymena*. In these four modules, no enrichment of biological functions are found in modules-13 and -20; module-8 shows a few enrichment terms related to phosphorylation and translation (Table S1), while the functions of module-14 may involve DNA repair, DNA replication and DNA integration etc. (Table S1). The enrichment functions are however likely not representative of the main functions of these modules, since the low homolog number leads to a few genes annotated by GO using BLAST based method in *Tetrahymena* (see Materials and Methods).

We have also investigated the overrepresented GO categories of other modules. Some modules such as module-3 (primary metabolic process) and module-4 (transport involved) show relatively singular functions, (Table S1). Others like modules-1 and -2 are involved with a group of related functions. This analysis will assist in understanding the functional clusters of genes and proteins in the ciliate *Tetrahymena*.

### Functional central genes in *Tetrahymena*

**Figure 4:**
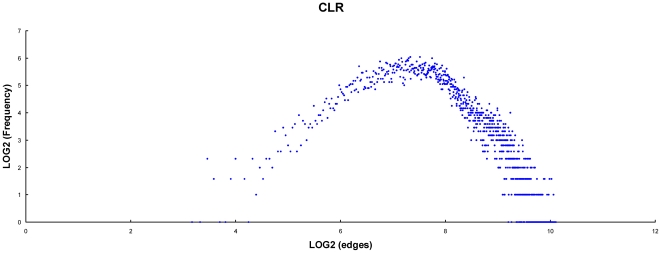
The distribution of the gained partners number of genes in TGN. The X-axis indicates the gained partners (each represents an edge) of genes, the value was Log2 transformed. The Y-axis indicates the frequency of gained partners number, also *Log2* transformed.

**Figure 5:**
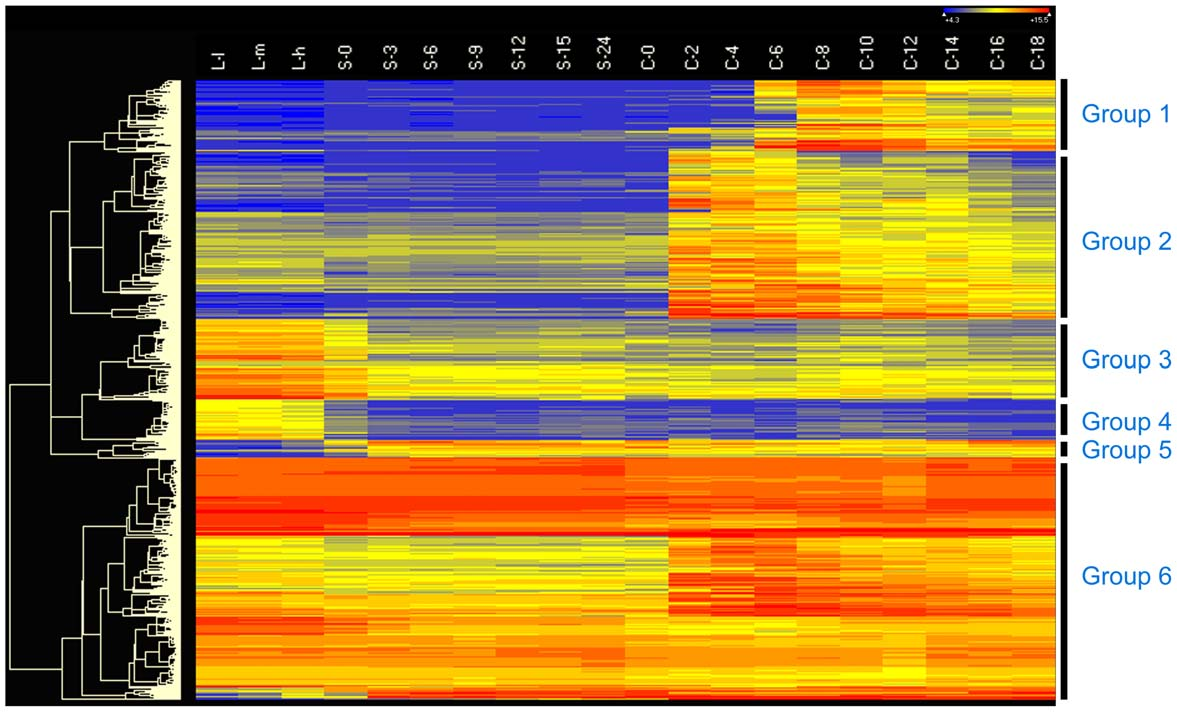
The heatmap of the 1200 hub genes in TGN. The heatmap was clustered by Euclidean distance of expression. The levels of expression are illustrated by different grades of color as determined from microarray data indicated along the top (from left to right). The color scale is as follows: dark color, low expression; light color, high expression. Levels of expression were obtained for 20 points in time during three physiological/developmental stages of the life cycle of *Tetrahymena*: For growing cells, L-l, L-m and L-h correspond to ∼1×10^5^ cells/ml, ∼3.5×10^5^ cells/ml and ∼1×10^6^ cells/ml, respectively. For measurements of expression during starvation, ∼2×10^5^ cells/ml were collected at intervals of 0, 3, 6, 9, 12, 15 and 24 hours (referred to as S-0, S-3, S-6, S-9, S-12, S-15 and S-24, respectively). For measurements of expression during conjugation, equal volumes of B2086 and CU428 cells were mixed following 18 h of starvation, and samples were collected at intervals of 0, 2, 4, 6, 8, 10, 12, 14, 16 and 18 h after mixing (referred to as C-0, C-2, C-4, C-6, C-8, C-10, C-12, C-14, C-16 and C-18, respectively). The 1,200 genes were sorted into six groups according to clustering analysis.

To better understand these functional centers, we investigated the distribution and the node degree of the TGN. The distribution of the node and edge number is shown in Figure 4. A power law tail of this distribution demonstrating that some of the genes in the network are highly connected with others, indicates that the network is scale free. This suggests that there are some hubs in the TGN. We have arbitrarily defined the top 1200 high connectively genes as hubs of the TGN (see Materials and Methods). These 1200 hubs can be sorted into 6 groups according to their expression profiles (Figure 5). Table S2 shows the detailed information and enrichment functions of the 6 groups.

**Group2** represents 326 hubs, which show specific high expression levels in the early stages of *Tetrahymena* conjugation. With 31.6 % of GO annotated genes, the overrepresentation functions are involved in a series of nuclear events, including DNA replication, DNA recombination, DNA repair and chromatin organization processes (Table S2), which are important events during early conjugation in *Tetrahymena*.

**Group4** contains 78 hubs specifically expressed during growth. Twenty-seven genes are annotated by GO terms and enrichment analysis shows 12 of them are overrepresented with two low level GO terms cofactor metabolic processes and cellular biosynthetic processes (Table S2). Twenty-one genes belong to a general high level GO term (metabolic processes) with an FDR value 8.1E-3. These data support that these genes are important for *Tetrahymena* growth.

Group6 contains 466 hub genes with 34.78 % annotated by GO terms. This group of genes has a continuous moderate or high expression level in all the stages of the *Tetrahymena* life cycle. Enriched GO terms indicate that these hubs are involved in not only basic cellular process such as DNA replication, transcription and translation (Table S2) but also in cellular metabolic processes such as glycolysis (GO:0006096) and the tricarboxylic acid cycle (GO:0006099) (data not shown). In addition, cellular biosynthetic processes (GO:0044249) are also over-represented with an FDR value 7.21E-6 (Table S2). These results suggest that these 466 hubs are essential for determination of the life cycle of this ciliate.

**Group1, Group3 and Group5**, shows no overrepresented and no significant GO terms (FDR value ∼0.05) in the enrichment analysis (Table S2). For the 1200 hub genes selected, about 35 % (Group1 and Group2) are specifically expressed in conjugation, 6.5 % (Group4) are specifically expressed in growth, and 38.8% (Group6) are continuously, moderately, or highly expressed in growth, starvation, and conjugation. However, no hub genes are found specifically expressed during starvation.

A hub of the scale-free network is very important and usually dominates the topology of the network .We have mapped the 1200 hubs to the modules that partition by the MCL method. Most of these hubs map intensively into the three biggest modules. The group1 and group2 hubs show the conjugation up-regulated expression pattern, and most of these are included in module-1 with overlapped GO enrichment terms (Table S2). The group3 and group4 hubs that dominate module-2 (Table S2) show growth up-regulated expression patterns, The group5 hubs are contained in module-3 with few genes. The group6 hubs, dispersedly map to four modules and overlap the enrichment GO terms with these modules (Table S2). Thus, the group6 hubs which express at continuous moderate or high level likely function throughout the *Tetrahymena* life cycle.

### Orthologs of human disease genes in *T. thermophila* but not in yeast

Many human genes including human disease genes have homologs or orthologs in model organisms where they can be readily studied. The yeast, *S. cerevisiae*, is a useful unicellular model organism, which can be used to study human genes involved in disease [46]. Many human disease genes are however not found as orthologs in this model organism. *Tetrahymena*, although phylogenetically distant from human, have many examples of genes found in human but not in yeast [19], [23]. Comparison between human and *Tetrahymena* shows that there are 58 orthologs (54 in TGN) of human disease genes in *Tetrahymena* but not in yeast [19]. We have extracted and analyzed the partners of each of these 54 genes from the TGN (Table S3). GO enrichment analysis suggests the potential use of these *Tetrahymena* genes for studying human disease genes (Table S3). Two cases are presented below:

Retinoblastoma, an embryonic malignant neoplasm of retinal origin, presents in early childhood and is often bilateral. The retinoblastoma (RB) gene was the first tumor suppressor gene cloned. It has been reported that this gene is closely related with cell cycle processes [47], [48], [49] and with DNA damage response pathways [50], [51]. The RB gene functionally interacts with components of the cell cycle machinery [52] and is phosphorylated by cyclin dependent kinases [53], [54], [55]. In addition, the RB gene is also related to ABC transporter genes [56], to minichromosome maintenance (MCM) genes [56] and to the transcription regulatory protein SNF2 gene [57], [58]. In *Tetrahymena*, there is an ortholog (TTHERM_00439030) of the human RB gene. This gene has 519 partners in TGN and 231 are annotated by GO terms. The overrepresented GO terms suggest that this gene with the partners identified may be involved in cell cycle and DNA-related metabolic processes, such as the cell cycle process (GO:0022402), regulation of cell cycle (GO:0051726), and DNA repair (GO:0006281) (Table S3). For the *Tetrahymena* ortholog of the human RB gene, we find cyclin genes, kinase genes, ABC transporter genes, MCM genes and SNF2 genes connected with TTHERM_00439030 (Table S4). We also find that this *Tetrahymena* RB ortholog is significantly related to histone proteins, identifying with the GO terms DNA packaging (GO:0006323) and chromatin organization (GO:0006325) in TGN (Table S3 and S4). This finding is consistent with reports that RB can recruit histone methyltransferase [59] and histone deacetylase [60]. These results suggest that the RB ortholog in *Tetrahymena* may play a similar role to the human retinoblastoma gene at the molecular level. In addition, the structural maintenance of chromosomes (SMC) family genes and the kinesin motor domain containing genes are also significantly related to the *Tetrahymena* RB ortholog (Table S4), suggesting these genes are likely functional relateded in the retinoblastoma pathway.

Another case of human disease gene is the NADH-ubiquinone oxidoreductase flavoprotein 1 (NDUFV1),which encodes a 51 kD subunit of complex I of the mitochondrial respiratory chain, and mutation of this gene leads to a mitochondrial complex I deficiency in human [61], [62]. As this gene belongs to complex I of electron transport, it should have many partners in the oxidative phosphorylation pathway. The *Tetrahymena* ortholog (TTHERM_00193910) of the human NDUFV1 gene has 347 partners in TGN. GO enrichment analysis results show these genes are involved in oxidative phosphorylation related terms: oxidation reduction, electron transport chain, tricarboxylic acid cycle, and hydrogen transport (Table S3). This result again suggests similar function between the human NDUFV1 gene and the *Tetrahymena* ortholog.

## Discussion

Physiological processes such as transcription, translation and metabolism evolve both within and between cells. To understand these dynamic processes, insight into interactions and combinations of independent genes and events is required. Constructing gene networks is a useful way to understand these physiological processes, and has been widely used in many common model organisms [6], [13], [15], [63], [64]. Based on machine learning methodology, gene network inference methods fall into two categories, supervised and unsupervised. Supervised methods start from a set of known interactions, and using this predefined training set evaluate new candidate genes as potential targets [65], [66]. Unsupervised methods do not use information from known network interactions [67], [68]. The method to be used depends on the datasets available and unsupervised methods are more suitable to infer the gene networks in some organisms[69], such as the ciliate protozoan *Tetrahymena*.

We report here the use of data from 67 expression microarrays to construct the *Tetrahymena* gene network using the unsupervised methods PCC, SCC and the CLR method. With the paucity of experimentally determined interactions in *Tetrahymena*, we chose four evolutionarily conserved protein complexes of yeast to validate the inferred networks. The CLR network performed with high accuracy and coverage with half of the total edges of correlation networks. To determine an appropriate cutoff confidence level of the CLR network, we chose the point of the F-score curve reaching a plateau at decreasing accuracy and increasing coverage. Faith et al. have reported 60% as the best cutoff level in the analysis of regulatory networks in *Escherichia coli* [26]. In the four protein complexes analyzed in the studies reported here, the minimal confidence level is 77%. Since the genes in protein complexes have a more coincident expression pattern than other interactions such as regulation and genes in same pathway, we chose the minimal 77% for the cutoff for the TGN.

After determining the appropriate cutoff confidence level, we used an efficient graphical clustering algorithm to partition the genome-wide TGN into gene modules based on the topological properties of the network. Genes in the same module are densely connected and provide a meaningful template for understanding biological processes. The GO enrichment analysis provides overrepresented terms of each module and indicates related biological pathways. Two examples of this analysis are: 1) *Tetrahymena* has separate germline and soma functions that are embodied by distinct nuclei within a single cell [18]. DNA rearrangement occurs during the programmed development of the new somatic macronucleus [70]. Module-1, although containing 3533 genes, is significantly enriched in genes related to this process. Using TGN to predict interactions involving eleven experimentally identified genes involved in developmentally programmed genome reorganization indicates a complex process involving many more genes than those identified to date. The analysis reported here provides a basis for further experimental analysis of developmental genome reorganization in *Tetrahymena*.

2) Oxidative phosphorylation is an important process in cellular respiration. In module-2, we have detected genes involved in this process, including components of the four complexes in the electron transport chain and ATP synthase including a unique ATP synthase [42]. We also found genes in related energy producing pathways such as glycolysis and the citric acid cycle to be closely connected to the oxidative phosphorylation genes.

We have designated 1200 genes in TGN as hubs based on connection or interaction number. Although a commonly held view is that hub nodes tend not to link to each other [30], we have found these hubs could be grouped by the expression patterns. The GO enrichment analysis for these hub groups shows that the overrepresented terms significantly relate to the expression of some hub groups. These results indicate some central genes playing important roles in different stages of the *Tetrahymena* life cycle. No hub genes were, however, found to be specifically expressed in the starvation stage of the *Tetrahymena* life cycle. Although starvation is an abnormal physiological condition providing an explanation for the absence of starvation-specific hub genes, genes expressed in both starvation and conjugation are found because starvation is required to induce conjugation [18]. The hubs are more concentrated in the large modules. With high connectivity, these genes dominate the structures of these modules, and also determine the related functions of the modules.

*Tetrahymena* is a unicellular microbial eukaryotic model organism with facile genetic manipulation. *Tetrahymena* has a high gene number and has more orthologs to human than to yeast [19]. We have analyzed the connected partners in TGN of 54 orthologs of human disease genes found in *Tetrahymena* but not in yeast. GO enrichment analysis shows that these orthologs and their interactions are likely to be involved in similar processes in human and *Tetrahymena.* Retinoblastoma is a rapidly developing cancer associated with mutation of the RB gene in humans. The RB gene has been extensively studied in human, and many experimentally determined interactions have illuminated involvement of the RB pathway in a number of biological processes. Through analysis of the partners of the *Tetrahymena* ortholog of the human RB gene, we found a very similar pattern of interacting genes in our constructed network. This suggests that *Tetrahymena* is potentially useful as a model to study molecular mechanisms of human disease genes.

## Materials and Methods

### Microarray data and gene filtering

The data used in this work correspond to a set of genome wide expression microarrays hybridized with mRNA samples coming mainly from growth, starvation and conjugation stages of the *Tetrahymena* life cycle [25]. A total of 67 NimbleGen single channel microarrays were collected and used (Table S5). Raw data are deposited in the Gene Expression Omnibus database, under accession numbers GSE11300, GSE26384, GSE26385 and GSE26650.

In order to remove genes with low information content, a combined filter criterion was used [15]. Based on between sample variability and gene-minimal signal, the filter leaves out only those gene that fulfilled both of the two following conditions: 1) Genes which have an expression difference or variability between samples (Exp_highest-lowest_) lower than the median of all the expression differences calculated for each gene (Exp_highest-lowest_<median Exp_highest-lowest_); 2) Genes which have a mean expression signal between samples lower than the median of all the expression signals calculated for each gene. After this filter, 12,973 genes were removed and 15,091 genes were used to construct the gene network.

### Network construction and validation

After the filtering, the expression values for the remaining 15,091 genes were *log*_2 _transformed. Three methods were used to construct the TGN, including the context likelihood of relatedness (CLR) algorithm [26], Pearson correlation coefficient (PCC), and Spearman correlation coefficient (SCC). For the CLR algorithm, the FDR test was performed to determine the confidence level for the Z-score.

For determining which method is the best and an appropriate threshold for the network, we have adopted the protein complex data of yeast to validate the network. The yeast protein complex data were downloaded from the CYC2008 [71], which provides an up-to-date reference set of both experimentally and computationally identified yeast protein complexes. We converted these yeast protein complexes to the *Tetrahymena* protein complexes based on the one to one orthologs. Since many of the converted *Tetrahymena* protein complexes are only a few genes, only connections for the four largest protein complexes in YeastNet v. 2 (http://www.yeastnet.org/) were used for the validation analysis as the “true positive” connections (edges). The performance was evaluated at different correlation coefficients and confidence levels, and three parameters, accuracy, coverage and overall performance were used to infer the performance [72]:

- The accuracy represents the percentage of inferred connections which are correct, defined as the p value.

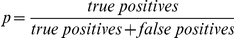

- The coverage represents the percentage of true connections that are correctly inferred by each method, defined as the r value.

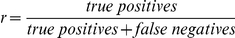

- The overall performance, called F-score, represents the compromise between *p* and *r* value, defined as follows:

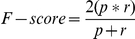



The nodes and edges under different correlation coefficients (PCC and SCC) or Z-score (CLR), the distribution of node degree and the validation are calculated using homemade Perl scripts.

### Ortholog retrieving

The OrthoMCL-DB Version 3 including 128 genomes was downloaded from the OrthoMCL website [73]. One to one orthologs between *Tetrahymena* and 9 other eukaryotes were extracted using homemade Perl script. Since no ortholog information between *Tetrahymena* and *Paramecium* exist in the database, the orthologs between *T. thermophila* and *P. tetraurelia* were determined by reciprocal best hit from a BLAST analysis. We selected a total of 10 eukaryotes based on evolutionary diversity, including *Homo sapiens*, *Danio rerio*, *Drosophila melanogaster*, *S. cerevisiae*, *Arabidopsis thaliana*, *Dictyostelium discoideum*, *Giardia lamblia*, *Plasmodium falciparum, Trypanosoma brucei,* and *P. tetraurelia.*

### Modules, hubs and orthologs of human disease genes

**Modules.** We used the MCL algorithm to partition TGN into gene modules [28]. The MCL software has an important parameter, named –I flag. This parameter has been evaluated to identify yeast protein complexes in protein-protein interaction networks using 1.8 (for –I flag) as the optimal value for the network [74]. Mao et al. [30] also used the 1.8 –I flag value to partition the *Arabidopsis *gene coexpression network with a set of 16,293 selected genes. In our work, we also chose 1.8 as the optimal value for *-*I flag to partition TGN using the MCL software.

To investigate the relationship between the modules and pathways, we extracted the KEGG pathway information from the KEGG Pathway Database website (http://www.genome.jp/kegg/pathway.html), and each pathway was matched to the modules. There are 90 *Tetrahymena* pathways in KEGG, 58 of them intensively matched to one module or to several modules (Table S1). Others are small pathways with few genes and matched to many modules.

**Hubs.** A clear definition of a hub protein in terms of the number of interacting partners, is not well-established, and the definition might vary from one dataset to another. Chad et al. [75] somewhat arbitrarily chose ten partners as a cutoff value and defined proteins with ≥10 partners as *hubs* in their work. Ashwini et al. [76] chose genes with more than five interactions as hubs, while Lu et al. [77] defined genes as nodes with connectivity greater than 5. In addition, Mao et al. [30] used the top 382 genes with at least 889 co-expression links as hubs in an *Arabidopsis* gene coexpression network. We chose the top 1,200 connected genes (about 5% of all *Tetrahymena* predicted proteins) as the hubs of TGN, and each of these 1,200 genes has at least 541 partners in TGN. The heatmap of these 1200 hubs was generated using the Euclidean distance as the cluster method in ArrayStar version 2.0 (DNASTAR, Inc, Madison, WI).

**Orthologs of human disease genes.** 58 *Tetrahymena* orthologs (54 in TGN) of human disease genes but not in yeast [19] were analyzed. The partners of each of the 54 genes in TGN were extracted to perform GO enrichment analysis.

### GO enrichment analysis

The gene ontology annotation was performed using the BLAST-based software Blast2GO for all predicted proteins of *Tetrahymena* as a reference. For the modules, groups, and disease related genes, the test gene set, GO term enrichment analysis was carried out by using Blast2GO. The false discovery rate (FDR) correction was used to control the false positive rate. If a GO term in a module showed an FDR corrected p value less than 0.05 in comparison with the reference, then the GO term was determined to be significantly enriched in the test gene set.

### Identification of Proteasomal Proteins

C-terminal tandem affinity tagged Dss1-FZZ plasmids were constructed as described by Witkin et al. [78]. DSS1 is the human homolog of the yeast proteasomal component Sem1 [79]. The *Tetrahymena* homolog of a protein annotated as a member of the Dss1/Sem protein family is TTHERM_00227230 (*Tetrahymena* Genome Database, http://www.ciliate.org; Rpn15).

*Tetrahymena* strain B2086 was biolistically transformed [80] with the Dss1-FZZ construct. Extracts were prepared from exponentially growing cells at 2×10^5^ cells/ml as described by Witkin et al. [78]. Dss1-Fzz and associated proteins were purified first on IgG-Sepharose (Amersham), eluted with TEV protease and subsequently immunopurified on M2-agarose (Sigma Chemical Co.) before being eluted with 3xFLAG peptide (Sigma). Silver stained bands from a 10% SDS PAGE gel were cut out, stored in 1% acetic acid and analyzed by MALDI mass spectrometry as described in Bowman et al. [81]. *Tetrahymena* proteasomal proteins identified were compared with yeast and human proteasomal proteins [79] and correlated with the *Tetrahymena* proteasome as presented on the KEGG web site (http://www.genome.jp/kegg/pathway.html).

## Supporting Information

Figure S1 **The accuracy, coverage and overall performance against the cutoff values (X-axis) of three methods for four protein complexes.** Blue, the accuracy, represented by p-value; pink, the coverage, represented by r-value; yellow, the overall performance, represented by F-score. For the CLR method, the cutoff value indicates the different confidence levels of the FDR test; for the PCC and SCC methods, the cutoff value represents the correlation coefficient.
(TIF)

Figure S2 **The network of genes very likely involved in MAC development.** Top represents the network of 11 experimentally identified genes involved in MAC development. The line width indicates the Z-score (also listed in the middle of the line). Bottom, the network of genes interacting with the 11 genes, representing 158 genes in total including the upper 11 genes, green square.
(TIF)

Figure S3 **The network of genes very likely involved in the ATP synthase processes.** Top represents the network of 71 genes of the ATP synthase complex identified by Mass Spectrometry [42]. Bottom is the network of 66 genes interacting with at least 60 of the upper 71 genes, representing a total of 137 genes including the upper 71 genes, green square.
(TIF)

Figure S4 **The corrected gene model and expression profile of TTHERM_01014660 (Rpt2).** A, an incorrectly predicted gene model of TTHERM_01014660. Red box, the corrected gene model determined by RNA-Seq, and five of fourteen microarray probes was located in the new gene model; B, comparison of the previous and re- normalized expression profile of TTHERM_01014660 in the *Tetrahymena* life cycle. Red, original normalization; Blue, re-normalized using the corrected gene model with five probes, the re-normalized expression profile is very similar to the other genes in the 19S proteasome regulatory particle (data not shown). For growing cells, **L-l**, **L-m** and **L-h** correspond respectively to ∼1×10^5^ cells/ml, ∼3.5×10^5^ cells/ml and ∼1×10^6^ cells/ml. For starvation, ∼2×10^5^ cells/ml were collected at 0, 3, 6, 9, 12, 15 and 24 hours(referred to as **S-0, S-3, S-6, S-9, S-12, S-15** and **S-24**). For conjugation, equal numbers of B2086 and CU428 cells were mixed after 18 h of starvation, and samples were collected at 0, 2, 4, 6, 8, 10, 12, 14, 16 and 18 hours after mixing (referred to as **C-0,** **C-2, C-4, C-6, C-8, C-10, C-12, C-14, C-16** and **C-18**) [25].
(TIF)

Table S1 **The enrichment functions of the 21 modules containing more than 100 genes in the TGN.** MF, molecular function; BP: biological process.
(XLS)

Table S2 **The enrichment functions of the 6 groups of hub genes.** GO ANNO NO, indicates the number of GO annotated genes; GO ANNO PER indicates the percentage of GO annotated genes; MF, molecular function; BP, biological process.
(XLS)

Table S3 **The partners of 56 orthologs of human disease genes and the enrichment functions.** GO ANNO NO, indicates the number of GO annotated genes; BP, biological process; MF, molecular function.
(DOC)

Table S4 **The partners of the** ***Tetrahymena*** **ortholog (TTHERM_00439030) of human retinoblastoma gene.** These partners were extracted from TGN with Z-score > 3.49 using TTHERM_00439030 as the bait, and the annotation of these partners were retrieved from TGD.
(XLS)

Table S5 **The information of the collected microarrays.** The three stages of the *Tetrahymena* life cycle involved in growth, starvation and conjugation. For growing cells, **L-l**, **L-m** and **L-h** correspond respectively to ∼1×10^5^ cells/ml, ∼3.5×10^5^ cells/ml and ∼1×10^6^ cells/ml. For starvation, ∼2×10^5^ cells/ml were collected at 0, 3, 6, 9, 12, 15 and 24 hours(referred to as **S-0, S-3, S-6, S-9, S-12, S-15** and **S-24**). For conjugation, equal numbers of B2086 and CU428 cells were mixed after 18 h of starvation, and samples were collected at 0, 15 min, 2, 4, 6, 8, 10, 12, 14, 16 and 18 hours after mixing (referred to as **C-0, C-15 m**, **C-2, C-4, C-6, C-8, C-10, C-12, C-14, C-16** and **C-18**). All 67 microarrays are highlighted based on GEO series. Red, GSE11300; Green, GSE26384; Blue, GSE26385; Purple, GSE26650.
(DOC)
