# Supplementary figures and images for: Gene Network Landscape of the Ciliate Tetrahymena thermophila
Source: PLoS One. 2011 May 26;6(5):e20124. doi: 10.1371/journal.pone.0020124 (PMC3102692; doi:10.1371/journal.pone.0020124)

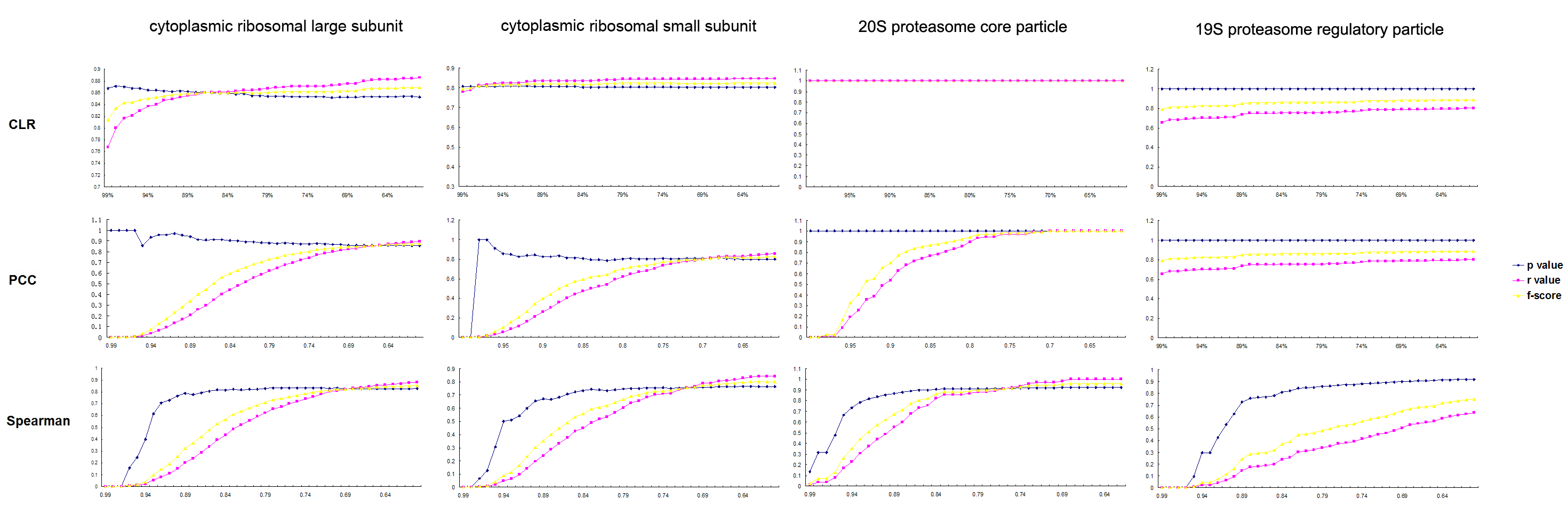

Supplement: Figure S1 — The accuracy, coverage and overall performance against the cutoff values (X-axis) of three methods for four protein complexes. Blue, the accuracy, represented by p-value; pink, the coverage, represented by r-value; yellow, the overall performance, represented by F-score. For the CLR method, the cutoff value indicates the different confidence levels of the FDR test; for the PCC and SCC methods, the cutoff value represents the correlation coefficient. (TIF) [file pone.0020124.s001.tif]

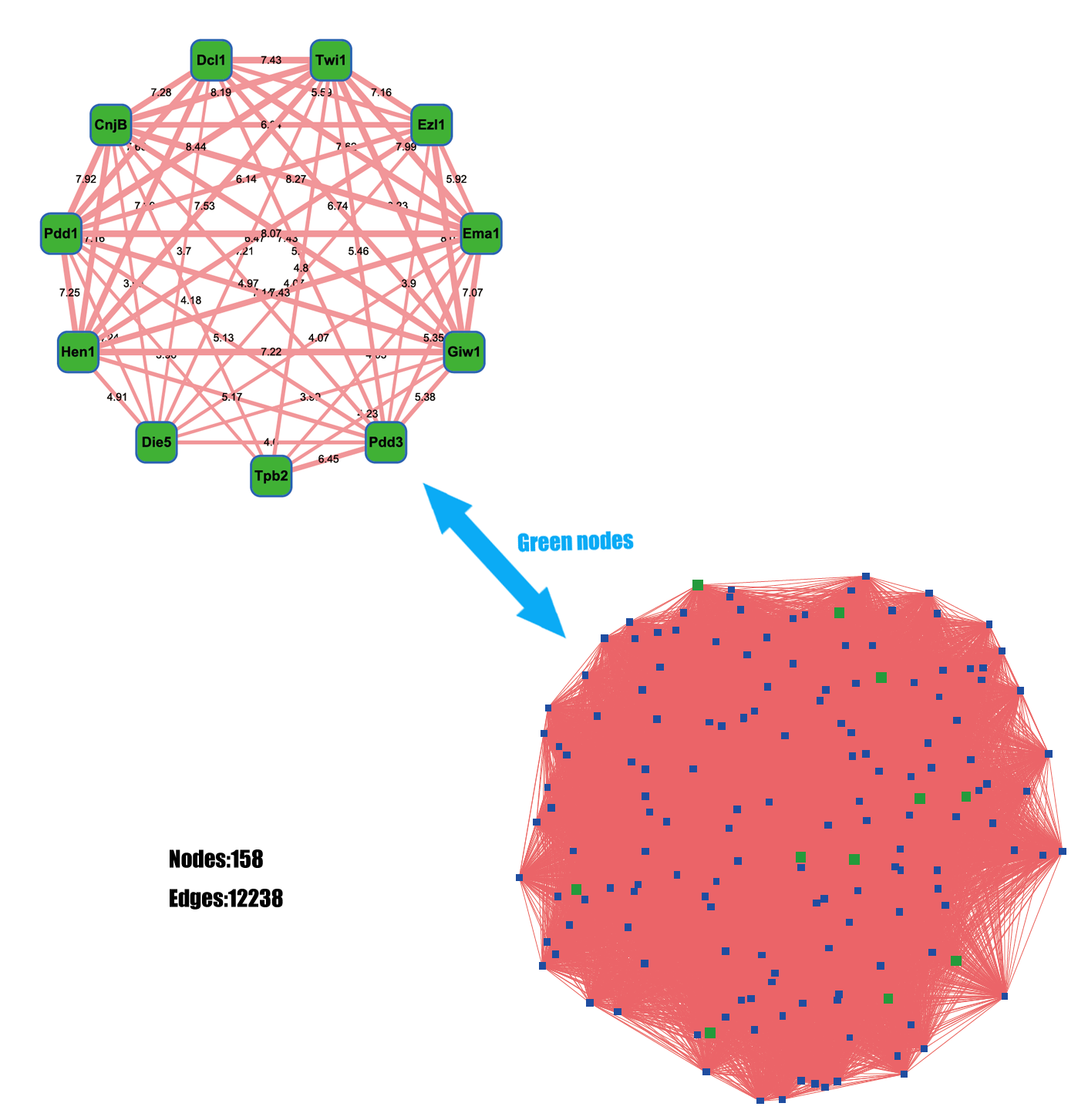

Supplement: Figure S2 — The network of genes very likely involved in MAC development. Top represents the network of 11 experimentally identified genes involved in MAC development. The line width indicates the Z-score (also listed in the middle of the line). Bottom, the network of genes interacting with the 11 genes, representing 158 genes in total including the upper 11 genes, green square. (TIF) [file pone.0020124.s002.tif]

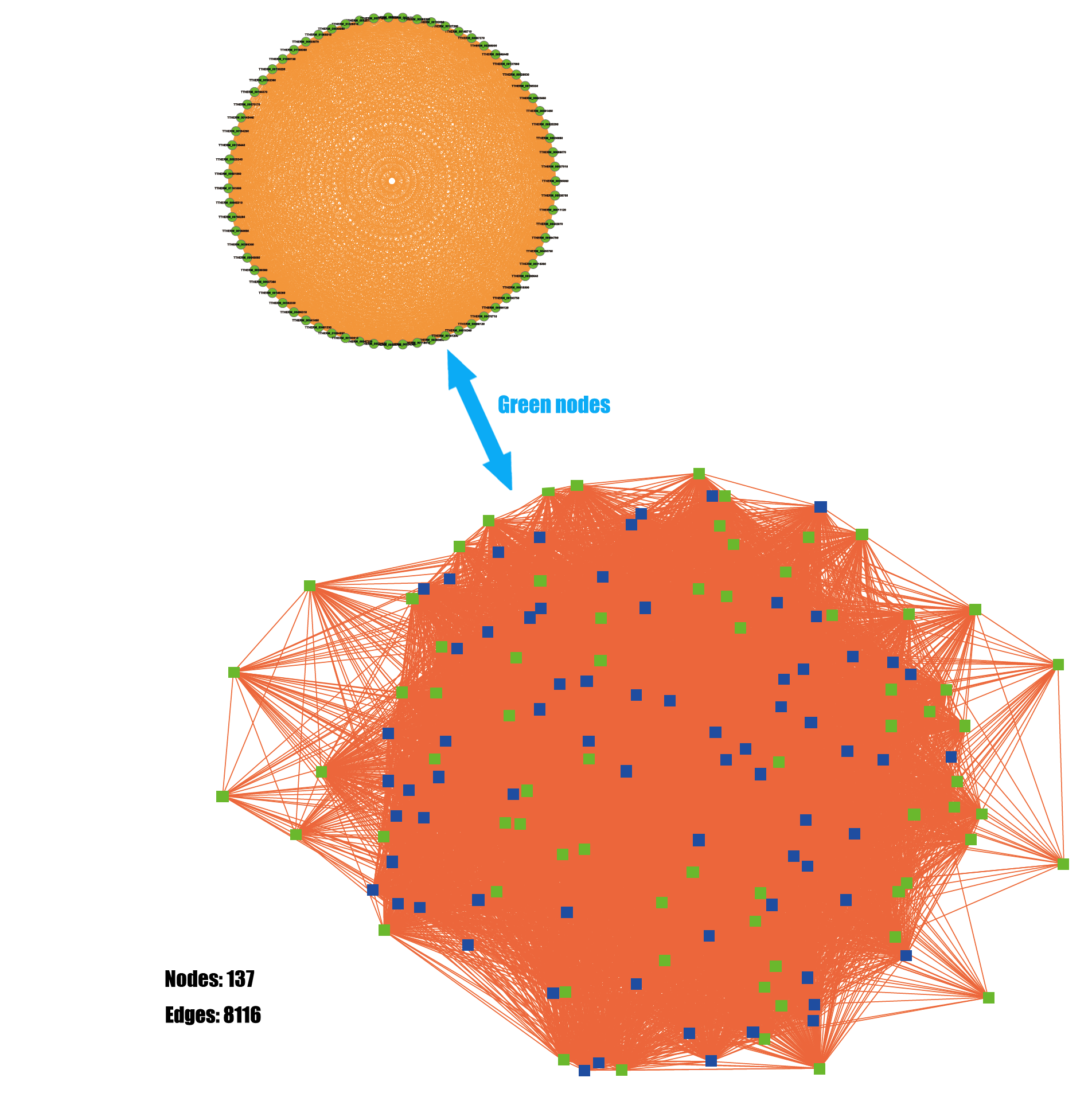

Supplement: Figure S3 — The network of genes very likely involved in the ATP synthase processes. Top represents the network of 71 genes of the ATP synthase complex identified by Mass Spectrometry [42]. Bottom is the network of 66 genes interacting with at least 60 of the upper 71 genes, representing a total of 137 genes including the upper 71 genes, green square. (TIF) [file pone.0020124.s003.tif]

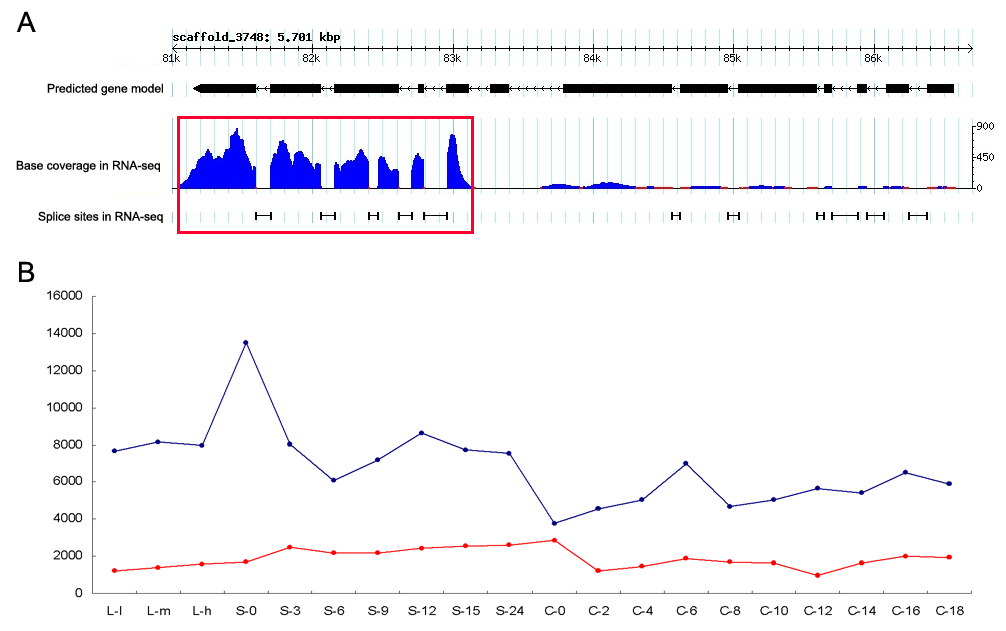

Supplement: Figure S4 — The corrected gene model and expression profile of TTHERM_01014660 (Rpt2). A, an incorrectly predicted gene model of TTHERM_01014660. Red box, the corrected gene model determined by RNA-Seq, and five of fourteen microarray probes was located in the new gene model; B, comparison of the previous and re- normalized expression profile of TTHERM_01014660 in the Tetrahymena life cycle. Red, original normalization; Blue, re-normalized using the corrected gene model with five probes, the re-normalized expression profile is very similar to the other genes in the 19S proteasome regulatory particle (data not shown). For growing cells, L-l, L-m and L-h correspond respectively to ∼1×10^5^ cells/ml, ∼3.5×10^5^ cells/ml and ∼1×10^6^ cells/ml. For starvation, ∼2×10^5^ cells/ml were collected at 0, 3, 6, 9, 12, 15 and 24 hours(referred to as S-0, S-3, S-6, S-9, S-12, S-15 and S-24). For conjugation, equal numbers of B2086 and CU428 cells were mixed after 18 h of starvation, and samples were collected at 0, 2, 4, 6, 8, 10, 12, 14, 16 and 18 hours after mixing (referred to as C-0, C-2, C-4, C-6, C-8, C-10, C-12, C-14, C-16 and C-18) [25]. (TIF) [file pone.0020124.s004.tif]
