# Supplementary material for: Gene Network Landscape of the Ciliate Tetrahymena thermophila
Source: PLoS One. 2011 May 26;6(5):e20124. doi: 10.1371/journal.pone.0020124 (PMC3102692; doi:10.1371/journal.pone.0020124)
Supplement: Table S5 — The information of the collected microarrays. The three stages of the Tetrahymena life cycle involved in growth, starvation and conjugation. For growing cells, L-l, L-m and L-h correspond respectively to ∼1×10^5^ cells/ml, ∼3.5×10^5^ cells/ml and ∼1×10^6^ cells/ml. For starvation, ∼2×10^5^ cells/ml were collected at 0, 3, 6, 9, 12, 15 and 24 hours(referred to as S-0, S-3, S-6, S-9, S-12, S-15 and S-24). For conjugation, equal numbers of B2086 and CU428 cells were mixed after 18 h of starvation, and samples were collected at 0, 15 min, 2, 4, 6, 8, 10, 12, 14, 16 and 18 hours after mixing (referred to as C-0, C-15 m, C-2, C-4, C-6, C-8, C-10, C-12, C-14, C-16 and C-18). All 67 microarrays are highlighted based on GEO series. Red, GSE11300; Green, GSE26384; Blue, GSE26385; Purple, GSE26650. (DOC) [file pone.0020124.s009.doc]

| **Stage** | **Time point** | **Microarray(s)** | **owner** | **GEO accession** |
| --- | --- | --- | --- | --- |
| Growth | L-l | 3 | MG and WM | GSM283687,GSM284355,GSM284362 |
|  | L-m | 3 | MG and WM | GSM283690,GSM284357,GSM284363 |
|  | L-h | 3 | MG and WM | GSM283691,GSM284360,GSM284364 |
| Starvation | S-0 | 3 | MG and WM | GSM285363,GSM285554,GSM285561 |
|  |  | 1 | WM | GSM647244 |
|  |  | 2 | YL | GSM647651,GSM647652 |
|  | S-3 | 3 | MG and WM | GSM285542,GSM285555,GSM285562 |
|  | S-6 | 3 | MG and WM | GSM285543,GSM285556,GSM285563 |
|  | S-9 | 3 | MG and WM | GSM285544,GSM285557,GSM285564 |
|  |  | 2 | YL | GSM647653,GSM647654 |
|  | S-12 | 3 | MG and WM | GSM285545,GSM285558,GSM285565 |
|  | S-15 | 3 | MG and WM | GSM285546,GSM285559,GSM285566 |
|  | S-24 | 3 | MG and WM | GSM285547,GSM285560,GSM285567 |
|  |  | 1 | WM | GSM647245 |
| Conjugation | C-0 | 2 | MG and WM | GSM285570,GSM285586 |
|  |  | 1 | REP | GSM656230 |
|  | C-15m | 1 | REP | GSM656231 |
|  | C-2 | 2 | MG and WM | GSM285572,GSM285587 |
|  |  | 1 | REP | GSM656233 |
|  | C-4 | 2 | MG and WM | GSM285574,GSM285588 |
|  |  | 1 | REP | GSM656234 |
|  | C-6 | 2 | MG and WM | GSM285575,GSM285589 |
|  |  | 1 | REP | GSM656232 |
|  | C-8 | 2 | MG and WM | GSM285576,GSM285590 |
|  |  | 1 | REP | GSM656236 |
|  | C-10 | 2 | MG and WM | GSM285578,GSM285591 |
|  |  | 1 | REP | GSM656235 |
|  | C-12 | 2 | MG and WM | GSM285579,GSM285592 |
|  |  | 1 | REP | GSM656237 |
|  | C-14 | 2 | MG and WM | GSM285580,GSM285593 |
|  |  | 1 | REP | GSM656238 |
|  | C-16 | 2 | MG and WM | GSM285582,GSM285595 |
|  |  | 1 | REP | GSM656239 |
|  | C-18 | 2 | MG and WM | GSM285583,GSM285596 |
|  |  | 1 | REP | GSM656240 |
| Total | / | 67 | MW (52), REP(11) and YL(4) | **/** |
