# Supplementary material for: Gene Network Landscape of the Ciliate Tetrahymena thermophila
Source: PLoS One. 2011 May 26;6(5):e20124. doi: 10.1371/journal.pone.0020124 (PMC3102692; doi:10.1371/journal.pone.0020124)
Supplement: Supplementary file 4 [file pone.0020124.s007.doc]

## Sheet: Sheet1

ORTHOLOG IN TETRAHYMENA	PARTNERS	GO ANNO NO	DISEASE DESCRIPTION 	Enriched BP	Enriched MF
TTHERM_00013890	191	86	Sucrose intolerance	GO:0005975 carbohydrate metabolic process GO:0006793 phosphorus metabolic process	GO:0016798 hydrolase activity, acting on glycosyl bonds GO:0016740 transferase activity GO:0016889 endodeoxyribonuclease activity, producing 3'-phosphomonoesters
TTHERM_00043770	108	38	Schindler disease	GO:0006508 proteolysis	GO:0016798 hydrolase activity, acting on glycosyl bonds GO:0004175 endopeptidase activity GO:0004536 deoxyribonuclease activity conclude:GO:0016787 hydrolase activity
TTHERM_00044990	241	109	Tyrosinemia, type I	GO:0009056 catabolic process GO:0042180 cellular ketone metabolic process GO:0006082 organic acid metabolic process GO:0006800 oxygen and reactive oxygen species metabolic process GO:0009853 photorespiration GO:0006519 cellular amino acid and derivative metabolic process GO:0042221 response to chemical stimulus GO:0034050 host programmed cell death induced by symbiont	GO:0016491 oxidoreductase activity GO:0016866 intramolecular transferase activity GO:0003746 translation elongation factor activity GO:0008266 poly(U) RNA binding GO:0008187 poly-pyrimidine tract binding GO:0016408 C-acyltransferase activity GO:0004372 glycine hydroxymethyltransferase activity
TTHERM_00046180	158	80	Tyrosinemia, type II	GO:0006082 organic acid metabolic process GO:0042180 cellular ketone metabolic process GO:0006807 nitrogen compound metabolic process GO:0040010 positive regulation of growth rate GO:0019439 aromatic compound catabolic process	GO:0048037 cofactor binding GO:0031420 alkali metal ion binding GO:0016769 transferase activity, transferring nitrogenous groups
TTHERM_00077350	95	41	Amyotrophic lateral sclerosis, juvenile	none	none
TTHERM_00128990	0	0	Pycnodysostosis	none	none
TTHERM_00136050	336	142	Fabry disease	GO:0006325 chromatin organization	GO:0016798 hydrolase activity, acting on glycosyl bonds
TTHERM_00137980	189	85	Mannosidosis, beta-	GO:0005975 carbohydrate metabolic process	GO:0016798 hydrolase activity, acting on glycosyl bonds
TTHERM_00138150	166	83	Glutaricaciduria, type I	GO:0006082 organic acid metabolic process GO:0042180 cellular ketone metabolic process GO:0055114 oxidation reduction GO:0044255 cellular lipid metabolic process GO:0051187 cofactor catabolic process GO:0006732 coenzyme metabolic process	GO:0016829 lyase activity GO:0016491 oxidoreductase activity GO:0048037 cofactor binding
TTHERM_00151140	114	33	Renal tubular acidosis-osteopetrosis syndrome	none	none
TTHERM_00193910	347	186	Mitochondrial complex I deficiency	GO:0009056 catabolic process GO:0005975 carbohydrate metabolic process GO:0065008 regulation of biological quality GO:0055114 oxidation reduction GO:0022900 electron transport chain  GO:0006099 tricarboxylic acid cycle  GO:0006818 hydrogen transport  GO:0009141 nucleoside triphosphate metabolic process GO:0051649 establishment of localization in cell GO:0009987 cellular process GO:0043648 dicarboxylic acid metabolic process GO:0046483 heterocycle metabolic process	GO:0003777 microtubule motor activity GO:0019842 vitamin binding GO:0048037 cofactor binding GO:0008641 small protein activating enzyme activity GO:0004774 succinate-CoA ligase activity GO:0042165 neurotransmitter binding GO:0042562 hormone binding GO:0016491 oxidoreductase activity GO:0051540 metal cluster binding GO:0016758 transferase activity, transferring hexosyl groups GO:0043498 cell surface binding GO:0009055 electron carrier activity GO:0008324 cation transmembrane transporter activity
TTHERM_00196650	274	152	Lactic acidosis due to defect in iron-sulfur cluster of complex I	GO:0006091 generation of precursor metabolites and energy GO:0005975 carbohydrate metabolic process	GO:0005509 calcium ion binding GO:0003824 catalytic activity GO:0008603 cAMP-dependent protein kinase regulator activity
TTHERM_00194260	239	98	Muscular dystrophy, limb-girdle, type 2A
TTHERM_00203010	527	220	Severe combined immunodeficiency, type I	GO:0006260 DNA replication GO:0006323 DNA packaging GO:0006325 chromatin organization GO:0043933 macromolecular complex subunit organization GO:0034641 cellular nitrogen compound metabolic process GO:0007049 cell cycle	GO:0019205 nucleobase, nucleoside, nucleotide kinase activity GO:0016763 transferase activity, transferring pentosyl groups GO:0016725 oxidoreductase activity, acting on CH or CH2 groups GO:0042801 polo kinase kinase activity GO:0003682 chromatin binding GO:0003676 nucleic acid binding
TTHERM_00214750	171	69	Oral-facial-digital syndrome 1	none	none
TTHERM_00220710	500	217	Phenylketonuria due to dihydropteridine reductase deficiency	GO:0006519 cellular amino acid and derivative metabolic process GO:0006082 organic acid metabolic process GO:0042180 cellular ketone metabolic process GO:0009058 biosynthetic process GO:0051186 cofactor metabolic process	GO:0016491 oxidoreductase activity GO:0004775 succinate-CoA ligase (ADP-forming) activity
TTHERM_00230960	185	77	Mannosidosis, alpha-	GO:0005975 carbohydrate metabolic process GO:0046466 membrane lipid catabolic process GO:0006026 aminoglycan catabolic process GO:0016998 cell wall macromolecule catabolic process	GO:0016798 hydrolase activity, acting on glycosyl bonds GO:0016298 lipase activity GO:0008234 cysteine-type peptidase activity
TTHERM_00285500	58	19	Sanfilippo syndrome, type B	none	none
TTHERM_00289080	414	233	Pyridoxine dependency with seizures	GO:0009058 biosynthetic process GO:0006807 nitrogen compound metabolic process GO:0042440 pigment metabolic process GO:0006519 cellular amino acid and derivative metabolic process GO:0006790 sulfur metabolic process GO:0033013 tetrapyrrole metabolic process GO:0051186 cofactor metabolic process GO:0055114 oxidation reduction GO:0009056 catabolic process GO:0006091 generation of precursor metabolites and energy conclude:GO:0044237 cellular metabolic process	GO:0048037 cofactor binding GO:0019842 vitamin binding GO:0031406 carboxylic acid binding GO:0003746 translation elongation factor activity GO:0016741 transferase activity, transferring one-carbon groups GO:0016829 lyase activity GO:0009055 electron carrier activity GO:0016491 oxidoreductase activity GO:0004830 tryptophan-tRNA ligase activity
TTHERM_00300520	200	99	Choroideremia	GO:0043632 modification-dependent macromolecule catabolic process GO:0051247 positive regulation of protein metabolic process GO:0051234 establishment of localization GO:0035148 tube formation GO:0007389 pattern specification process GO:0007264 small GTPase mediated signal transduction GO:0007224 smoothened signaling pathway GO:0009653 anatomical structure morphogenesis	GO:0003924 GTPase activity GO:0019783 small conjugating protein-specific protease activity GO:0005083 small GTPase regulator activity GO:0019001 guanyl nucleotide binding
TTHERM_00317200	422	187	Hemolytic anemia due to adenylate kinase deficiency	GO:0051186 cofactor metabolic process GO:0055085 transmembrane transport GO:0006818 hydrogen transport GO:0045454 cell redox homeostasis GO:0009056 catabolic process GO:0006066 alcohol metabolic process GO:0007017 microtubule-based process	GO:0005509 calcium ion binding GO:0050662 coenzyme binding GO:0019205 nucleobase, nucleoside, nucleotide kinase activity GO:0016491 oxidoreductase activity GO:0008324 cation transmembrane transporter activity
TTHERM_00321550	215	72	Mucopolysaccharidosis IVB	none	none
TTHERM_00325580	499	284	Maple syrup urine disease, type Ia	GO:0051186 cofactor metabolic process GO:0046483 heterocycle metabolic process GO:0006733 oxidoreduction coenzyme metabolic process GO:0009058 biosynthetic process GO:0008152 metabolic process GO:0042221 response to chemical stimulus	GO:0051082 unfolded protein binding GO:0019842 vitamin binding GO:0048037 cofactor binding GO:0031406 carboxylic acid binding GO:0016829 lyase activity GO:0016853 isomerase activity GO:0016741 transferase activity, transferring one-carbon groups GO:0016491 oxidoreductase activity
TTHERM_00363210	715	325	Progressive external ophthalmoplegia, type 2	GO:0008152 metabolic process GO:0034220 ion transmembrane transport GO:0006818 hydrogen transport	GO:0019842 vitamin binding GO:0051540 metal cluster binding GO:0003779 actin binding GO:0005506 iron ion binding GO:0015077 monovalent inorganic cation transmembrane transporter activity GO:0003824 catalytic activity GO:0009055 electron carrier activity
TTHERM_00439030	519	231	Retinoblastoma	GO:0048856 anatomical structure development GO:0006281 DNA repair  GO:0009058 biosynthetic process GO:0006807 nitrogen compound metabolic process GO:0030261 chromosome condensation  GO:0006323 DNA packaging  GO:0006325 chromatin organization  GO:0051726 regulation of cell cycle GO:0022402 cell cycle process GO:0007059 chromosome segregation	GO:0019205 nucleobase, nucleoside, nucleotide kinase activity GO:0016779 nucleotidyltransferase activity GO:0016725 oxidoreductase activity, acting on CH or CH2 groups GO:0008094 DNA-dependent ATPase activity GO:0003676 nucleic acid binding GO:0005515 protein binding GO:0003682 chromatin binding
TTHERM_00440600	207	57	Muscular dystrophy, limb-girdle, type 2B	GO:0019932 second-messenger-mediated signaling GO:0006753 nucleoside phosphate metabolic process GO:0055086 nucleobase, nucleoside and nucleotide metabolic process	GO:0016787 hydrolase activity GO:0003746 translation elongation factor activity GO:0005516 calmodulin binding GO:0019239 deaminase activity
TTHERM_00442180	410	219	Ketoacidosis due to SCOT deficiency	GO:0008152 metabolic process GO:0042221 response to chemical stimulus GO:0048534 hemopoietic or lymphoid organ development GO:0016265 death GO:0065008 regulation of biological quality	GO:0019842 vitamin binding GO:0048037 cofactor binding GO:0003746 translation elongation factor activity GO:0008187 poly-pyrimidine tract binding GO:0008266 poly(U) RNA binding GO:0003824 catalytic activity GO:0051540 metal cluster binding GO:0009055 electron carrier activity
TTHERM_00442280	0	0	Retinitis pigmentosa, autosomal recessive	none	none
TTHERM_00444520	0	0	Polycystic kidney disease, adult, type II	none	none
TTHERM_00445920	224	54	Papillon-Lefevre syndrome	GO:0044271 cellular nitrogen compound biosynthetic process GO:0034654 nucleobase, nucleoside, nucleotide and nucleic acid biosynthetic process GO:0009117 nucleotide metabolic process	GO:0003746 translation elongation factor activity GO:0005516 calmodulin binding GO:0070001 aspartic-type peptidase activity GO:0019239 deaminase activity
TTHERM_00446240	227	117	Alkaptonuria	GO:0050896 response to stimulus GO:0034050 host programmed cell death induced by symbiont GO:0006082 organic acid metabolic process GO:0006519 cellular amino acid and derivative metabolic process GO:0006800 oxygen and reactive oxygen species metabolic process GO:0006807 nitrogen compound metabolic process GO:0009056 catabolic process GO:0055114 oxidation reduction GO:0042180 cellular ketone metabolic process	GO:0016853 isomerase activity GO:0016769 transferase activity, transferring nitrogenous groups GO:0004372 glycine hydroxymethyltransferase activity GO:0016491 oxidoreductase activity GO:0016829 lyase activity GO:0019842 vitamin binding GO:0008187 poly-pyrimidine tract binding GO:0008266 poly(U) RNA binding
TTHERM_00463860	17	10	Bardet-Biedl syndrome 2	none	none
TTHERM_00471380	161	67	Stomatocytosis I	none	none
TTHERM_00476630	171	65	Mulibrey nanism	none	none
TTHERM_00487090	120	68	Aldolase A deficiency	GO:0055114 oxidation reduction GO:0009056 catabolic process GO:0005975 carbohydrate metabolic process GO:0006091 generation of precursor metabolites and energy GO:0051186 cofactor metabolic process GO:0006082 organic acid metabolic process GO:0042180 cellular ketone metabolic process GO:0006066 alcohol metabolic process	GO:0048037 cofactor binding GO:0019842 vitamin binding GO:0016853 isomerase activity GO:0004774 succinate-CoA ligase activity GO:0016829 lyase activity GO:0016491 oxidoreductase activity GO:0016757 transferase activity, transferring glycosyl groups
TTHERM_00494870	218	103	Spinal muscular atrophy, HEXB-related	GO:0044036 cell wall macromolecule metabolic process GO:0006027 glycosaminoglycan catabolic process GO:0005975 carbohydrate metabolic process	GO:0016798 hydrolase activity, acting on glycosyl bonds GO:0008242 omega peptidase activity
TTHERM_00526930	345	130	Complex I deficiency	GO:0051186 cofactor metabolic process GO:0046483 heterocycle metabolic process GO:0006818 hydrogen transport	GO:0051540 metal cluster binding GO:0000036 acyl carrier activity GO:0004775 succinate-CoA ligase (ADP-forming) activity
TTHERM_00535640	224	99	Glycogen storage disease II	none	none
TTHERM_00535830	108	33	Bardet-Biedl syndrome 3	GO:0010498 proteasomal protein catabolic process	none
TTHERM_00586610	121	50	Lowe syndrome	GO:0006793 phosphorus metabolic process GO:0044260 cellular macromolecule metabolic process GO:0019538 protein metabolic process GO:0043412 macromolecule modification	GO:0016772 transferase activity, transferring phosphorus-containing groups GO:0000166 nucleotide binding GO:0001883 purine nucleoside binding
TTHERM_00591650	280	107	Ceroid lipofuscinosis, neuronal-1, infantile	GO:0005975 carbohydrate metabolic process	GO:0016798 hydrolase activity, acting on glycosyl bonds GO:0008234 cysteine-type peptidase activity 
TTHERM_00678260	85	53	Tyrosinemia, type III (1);Corneal dystrophy, posterior polymorphous	GO:0016072 rRNA metabolic process GO:0044255 cellular lipid metabolic process GO:0019439 aromatic compound catabolic process GO:0006066 alcohol metabolic process GO:0042180 cellular ketone metabolic process GO:0006082 organic acid metabolic process	GO:0070279 vitamin B6 binding GO:0016408 C-acyltransferase activity GO:0016491 oxidoreductase activity 
TTHERM_00686100	249	108	HMG-CoA lyase deficiency	GO:0006414 translational elongation GO:0016054 organic acid catabolic process GO:0019752 carboxylic acid metabolic process GO:0009308 amine metabolic process GO:0009853 photorespiration GO:0006800 oxygen and reactive oxygen species metabolic process GO:0051186 cofactor metabolic process GO:0034050 host programmed cell death induced by symbiont GO:0065002 intracellular protein transmembrane transport GO:0007005 mitochondrion organization	GO:0016491 oxidoreductase activity GO:0008135 translation factor activity, nucleic acid binding GO:0003727 single-stranded RNA binding GO:0008187 poly-pyrimidine tract binding GO:0016829 lyase activity GO:0016741 transferase activity, transferring one-carbon groups
TTHERM_00691710	242	119	PEO with mitochondrial DNA deletions, type 1	GO:0065002 intracellular protein transmembrane transport GO:0006626 protein targeting to mitochondrion	GO:0016857 racemase and epimerase activity, acting on carbohydrates and derivatives GO:0008135 translation factor activity, nucleic acid binding GO:0016741 transferase activity, transferring one-carbon groups GO:0051082 unfolded protein binding
TTHERM_00704010	341	186	Maple syrup urine disease, type Ib	GO:0006457 protein folding GO:0009058 biosynthetic process GO:0009308 amine metabolic process GO:0055114 oxidation reduction GO:0051186 cofactor metabolic process GO:0006082 organic acid metabolic process GO:0042180 cellular ketone metabolic process GO:0042221 response to chemical stimulus GO:0016265 death GO:0045454 cell redox homeostasis GO:0006886 intracellular protein transport	GO:0008187 poly-pyrimidine tract binding GO:0048037 cofactor binding GO:0008266 poly(U) RNA binding GO:0051082 unfolded protein binding GO:0016417 S-acyltransferase activity GO:0016741 transferase activity, transferring one-carbon groups GO:0016835 carbon-oxygen lyase activity GO:0016491 oxidoreductase activity GO:0016853 isomerase activity
TTHERM_00794540	388	205	Maple syrup urine disease, type II	GO:0042180 cellular ketone metabolic process GO:0006082 organic acid metabolic process GO:0006519 cellular amino acid and derivative metabolic process GO:0055114 oxidation reduction GO:0009056 catabolic process GO:0051186 cofactor metabolic process GO:0050896 response to stimulus GO:0034050 host programmed cell death induced by symbiont	GO:0003727 single-stranded RNA binding GO:0051082 unfolded protein binding GO:0008187 poly-pyrimidine tract binding GO:0019842 vitamin binding GO:0048037 cofactor binding GO:0008135 translation factor activity, nucleic acid binding GO:0016491 oxidoreductase activity GO:0016829 lyase activity GO:0016853 isomerase activity GO:0016741 transferase activity, transferring one-carbon groups
TTHERM_00833870	182	53	Zellweger syndrome-3	none	none
TTHERM_00927280	167	74	Smith-Lemli-Opitz syndrome	none	none
TTHERM_00942830	0	0	Usher syndrome, type 2	none	none
TTHERM_01018250	288	82	Epidermolytic palmoplantar keratoderma	GO:0045449 regulation of transcription GO:0043170 macromolecule metabolic process GO:0006259 DNA metabolic process GO:0006139 nucleobase, nucleoside, nucleotide and nucleic acid metabolic process GO:0018193 peptidyl-amino acid modification GO:0006807 nitrogen compound metabolic process GO:0006974 response to DNA damage stimulus GO:0000160 two-component signal transduction system (phosphorelay)	GO:0016787 hydrolase activity GO:0008234 cysteine-type peptidase activity GO:0016788 hydrolase activity, acting on ester bonds GO:0016779 nucleotidyltransferase activity GO:0030528 transcription regulator activity GO:0016775 phosphotransferase activity, nitrogenous group as acceptor GO:0004673 protein histidine kinase activity GO:0003676 nucleic acid binding
TTHERM_01019590	86	39	Isovalericacidemia	none	GO:0015932 nucleobase, nucleoside, nucleotide and nucleic acid transmembrane transporter activity 
TTHERM_01054280	107	40	Bardet-Biedl syndrome 4	none	none
TTHERM_01084290	247	62	Hyperphenylalaninemia due to pterin-4a-carbinolamine dehydratase deficiency	GO:0006082 organic acid metabolic process GO:0042180 cellular ketone metabolic process GO:0055114 oxidation reduction GO:0006091 generation of precursor metabolites and energy GO:0044275 cellular carbohydrate catabolic process GO:0005996 monosaccharide metabolic process GO:0006732 coenzyme metabolic process GO:0015672 monovalent inorganic cation transport	GO:0015077 monovalent inorganic cation transmembrane transporter activity GO:0016721 oxidoreductase activity, acting on superoxide radicals as acceptor GO:0004738 pyruvate dehydrogenase activity GO:0016614 oxidoreductase activity, acting on CH-OH group of donors GO:0003755 peptidyl-prolyl cis-trans isomerase activity
TTHERM_01098980	128	49	Shprintzen-Goldberg syndrome	GO:0006812 cation transport GO:0006811 ion transport	GO:0022857 transmembrane transporter activity GO:0022892 substrate-specific transporter activity
TTHERM_01193550	523	235	Lysosomal acid phosphatase deficiency	GO:0006807 nitrogen compound metabolic process GO:0006519 cellular amino acid and derivative metabolic process GO:0043094 cellular metabolic compound salvage GO:0055114 oxidation reduction GO:0009058 biosynthetic process GO:0006629 lipid metabolic process GO:0006082 organic acid metabolic process GO:0042180 cellular ketone metabolic process GO:0010035 response to inorganic substance	GO:0019842 vitamin binding GO:0048037 cofactor binding GO:0003727 single-stranded RNA binding GO:0008187 poly-pyrimidine tract binding GO:0016491 oxidoreductase activity GO:0016829 lyase activity GO:0016741 transferase activity, transferring one-carbon groups

## Sheet: Sheet2

## Sheet: Sheet3
